# Reduction of Outdoor and Indoor PM_2.5_ Source Contributions via Portable Air Filtration Systems in a Senior Residential Facility in Detroit, Michigan

**DOI:** 10.3390/toxics11121019

**Published:** 2023-12-14

**Authors:** Zachary M. Klaver, Ryan C. Crane, Rosemary A. Ziemba, Robert L. Bard, Sara D. Adar, Robert D. Brook, Masako Morishita

**Affiliations:** 1 Exposure Science Lab, Family Medicine, College of Human Medicine, Michigan State University, East Lansing, MI 48824, USA; 2 Community Health Nursing, Ann Arbor, MI 48109, USA; 3 Division of Cardiovascular Medicine, University of Michigan, Ann Arbor, MI 48109, USA; 4 School of Public Health, University of Michigan, Ann Arbor, MI 48109, USA; 5 Department of Internal Medicine, Wayne State University, Detroit, MI 48201, USA

**Keywords:** indoor PM_2.5_, source apportionment, air filtration, cardiovascular health

## Abstract

**Background:** The Reducing Air Pollution in Detroit Intervention Study (RAPIDS) was designed to evaluate cardiovascular health benefits and personal fine particulate matter (particulate matter < 2.5 μm in diameter, PM_2.5_) exposure reductions via portable air filtration units (PAFs) among older adults in Detroit, Michigan. This double-blind randomized crossover intervention study has shown that, compared to sham, air filtration for 3 days decreased 3-day average brachial systolic blood pressure by 3.2 mmHg. The results also showed that commercially available HEPA-type and true HEPA PAFs mitigated median indoor PM_2.5_ concentrations by 58% and 65%, respectively. However, to our knowledge, no health intervention study in which a significant positive health effect was observed has also evaluated how outdoor and indoor PM_2.5_ sources impacted the subjects. With that in mind, detailed characterization of outdoor and indoor PM_2.5_ samples collected during this study and a source apportionment analysis of those samples using a positive matrix factorization model were completed. The aims of this most recent work were to characterize the indoor and outdoor sources of the PM_2.5_ this community was exposed to and to assess how effectively commercially available HEPA-type and true HEPA PAFs were able to reduce indoor and outdoor PM_2.5_ source contributions. **Methods:** Approximately 24 h daily indoor and outdoor PM_2.5_ samples were collected on Teflon and Quartz filters from the apartments of 40 study subjects during each 3-day intervention period. These filters were analyzed for mass, carbon, and trace elements. Environmental Protection Agency Positive Matrix Factorization (PMF) 5.0 was utilized to determine major emission sources that contributed to the outdoor and indoor PM_2.5_ levels during this study. **Results:** The major sources of outdoor PM_2.5_ were secondary aerosols (28%), traffic/urban dust (24%), iron/steel industries (15%), sewage/municipal incineration (10%), and oil combustion/refinery (6%). The major sources of indoor PM_2.5_ were organic compounds (45%), traffic + sewage/municipal incineration (14%), secondary aerosols (13%), smoking (7%), and urban dust (2%). Infiltration of outdoor PM_2.5_ for sham, HEPA-type, and true HEPA air filtration was 79 ± 24%, 61 ± 32%, and 51 ± 34%, respectively. **Conclusions:** The results from our study showed that intervention with PAFs was able to significantly decrease indoor PM_2.5_ derived from outdoor and indoor PM_2.5_ sources. The PAFs were also able to significantly reduce the infiltration of outdoor PM_2.5_. The results of this study provide insights into what types of major PM_2.5_ sources this community is exposed to and what degree of air quality and systolic blood pressure improvements are possible through the use of commercially available PAFs in a real-world setting.

## 1. Introduction

The adverse effects of ambient fine particulate matter (particulate matter < 2.5 μm in diameter, PM_2.5_) on cardiovascular (CV) health are well established [1,2,3,4,5]. As such, the American Heart Association and European Society of Cardiology, as well as the U.S. Environmental Protection Agency (EPA), have all recognized PM_2.5_ as a causal risk factor for CV diseases [6]. Despite a nationwide 41% reduction in PM_2.5_ over the last 20 years, over 26 million people are still living in areas with PM_2.5_ levels in excess of the National Ambient Air Quality Standards (NAAQS). In addition, some studies show that even PM_2.5_ levels below the NAAQS pose CV health risks [7,8,9]. This presents an urgent need to utilize methods of exposure reduction beyond governmental policies in order to protect susceptible populations.

There is a growing body of evidence that high-efficiency particulate arrestance (HEPA) filtration can reduce indoor PM_2.5_ concentrations and deliver CV health benefits via the reduction of exposure to PM [10,11,12,13,14,15,16]. Given that people spend approximately 90% of their time indoors [17,18], interventions targeted at reducing indoor PM_2.5_ concentrations could be a practical means of reducing overall personal PM_2.5_ exposure. The Reducing Air Pollution in Detroit Intervention Study (RAPIDS) was designed to evaluate CV health benefits and personal PM_2.5_ exposure reductions via portable air filtration units (PAFs) among older adults in Detroit, Michigan. This double-blind randomized crossover intervention study has shown that, compared to sham, air filtration for 3 days using PAFs decreased 3-day average brachial systolic blood pressure by 3.2 mmHg [19]. The results showed that HEPA-type and true HEPA PAFs mitigated median indoor PM_2.5_ concentrations by 39% and 50%, respectively [20]. However, it was still unknown what kinds of indoor and outdoor PM_2.5_ sources were impacting this study participants and how much outdoor PM_2.5_ infiltrated into indoor environments in the community, where multiple PM_2.5_ point and mobile sources are located.

In this paper, we describe the detailed chemical characterization of outdoor and indoor PM_2.5_ this community was exposed to, the results of source apportionment analysis using a positive matrix factorization model on the outdoor and indoor PM_2.5_ data, and how effectively commercially available high-efficiency (HE: true-HEPA) and low-efficiency (LE: HEPA-type) PAFs were able to reduce indoor and outdoor PM_2.5_ source contributions. The results of this work provide insights into what degree of air quality improvement is possible in one of the most vulnerable communities through the use of commercially available PAFs.

## 2. Methods

### 2.1. Intervention

This study enrolled 40 older adult subjects (age 67 ± 8 years) not receiving supplementary oxygen and living in a government-subsidized, low-income residential building for senior citizens in Midtown Detroit. Individual residences used the same floor plan (approximately 47 m^2^) and hydronic baseboard heating. This study was approved by the institutional review board of the University of Michigan, and participants signed a written informed consent document during screening visits. Details of this randomized double-blind crossover intervention study have been described previously [19]. In brief, we placed one PAF (model HAP424, Holmes, Milford, MA, USA) in the bedroom and one PAF in the main living space of each subject’s residence. The subjects underwent 3 separate intervention periods, each consisting of 3 days. During each intervention period, subjects were exposed to 3 blinded scenarios in random order: unfiltered air (no filter), LE (“HEPA type”, model HAPF30D-U2, Holmes, Milford, MA, USA), and HE (“True HEPA”, model HAPF300D-U2, Holmes, Milford, MA, USA). Intervention periods for each subject were separated by a washout period of at least one week.

### 2.2. Sampling

Approximately 24 h daily indoor and outdoor PM_2.5_ concentrations were measured during each 3-day intervention period. Indoor PM_2.5_ samples were collected onto 47 mm Teflon and quartz filters using a custom-built pump system consisting of an acoustically insulated wood case designed for operation in indoor environments, thus minimizing pump noise during sampling periods. Teflon-coated aluminum cyclone sample inlets (URG, Chapel Hill, NC, USA) with a nominal flow rate of 16.7 L/min to provide a 2.5 μm particle cutpoint and a calibrated rotameter (Matheson Inc., Montgomeryville, PA, USA) were used to verify the nominal flow rate. Outdoor PM_2.5_ samples were collected using a sequential air sampler (Partisol-Plus Model 2025, Rupprecht and Patashnick, Inc., Albany, NY, USA) located on the roof of a 3-story building 125 m from the residential facility. These outdoor PM_2.5_ samples were collected on 47 mm Teflon filters for subsequent gravimetric, black carbon (BC), and elemental analyses.

### 2.3. Mass and Chemical Analyses

Sample handling, processing, and analysis took place in Class 100 clean rooms at the Michigan State University Exposure Science Laboratory. Mass concentrations were determined gravimetrically using a microbalance (Model XPR6UD5, Mettler Toledo, Columbus, OH, USA) after the filters had been conditioned for 24 h in a temperature- and humidity-controlled environment. Indoor PM_2.5_ samples collected on quartz filters were analyzed for organic carbon (OC) and elemental carbon (EC) by the NIOSH 5040 method using a thermal-optical OC/EC analyzer (Model 5L, Sunset Labs, Tigard, OR, USA). Outdoor PM_2.5_ Teflon samples were analyzed for black carbon content via a SootScan Model OT21 Optical Transmissometer analyzer (Magee Scientific, Oxford, UK). This was a non-destructive technique that left the sample intact for subsequent trace metal analysis.

Concentrations of 36 trace elements were determined for outdoor and indoor samples by inductively coupled plasma mass spectrometry (ELEMENT2, Thermo Fisher Scientific, Waltham, MA, USA). The Teflon filters were extracted by an acid digestion process as described previously [21]. In brief, the Teflon filters were wetted with ethanol and placed in acid-cleaned 15 mL polypropylene centrifuge tubes containing a 1% nitric acid solution. These tubes were placed in a sonicator (Ultrasonic Bath CPX8800, Thermo Fisher Scientific, Waltham, MA, USA) for a continuous 48 h period, and the filters were then passively digested for 2 weeks before the extracts were analyzed. This analysis method incorporated daily quality assurance and quality control measures, including field blanks, Type-1 water blanks, replicate analyses, and external standards. Method detection limits (MDLs) were calculated as three times the standard deviation of seven consecutive measurements of a sample that falls approximately in the middle of the concentration distribution curve.

### 2.4. Data Analysis

Source apportionment was completed using 36 trace metal concentrations and uncertainties quantified via ICP-MS from 257 outdoor samples and 358 indoor samples. Major emission sources contributing to outdoor and indoor PM_2.5_ levels in Detroit were determined via the EPAs Positive Matrix Factorization (PMF) 5.0 Fundamentals and User Guide [22]. PMF is a multivariate factor analysis model based on the least squares fit and error estimation from a PM_2.5_ chemical dataset [23,24]. The analytical measurement uncertainty (*AM*), sample collection uncertainty (*SC*), and MDLs were used to calculate the uncertainty (*U*) assigned to each measured concentration data point as follows [22,25]:

$$U=\sqrt{{\left(\sqrt{{\left(SC\right)}^{2}+{\left(AM\right)}^{2}}*\left(concentration\right)\right)}^{2}+{\left(MDL\right)}^{2}}$$

For this study, *AM* was the relative standard deviation for each sample from the ICP-MS analysis. *SC* was estimated to be about 10%. Concentrations below the MDL were replaced by half of the MDL [25], and uncertainties were calculated using the equation above.

Based on the PMF 5.0 Fundamentals and User Guide and Paatero and Hopke, signal-to-noise ratios, poorly predicted values, and R^2^ were used to determine species categorization [22,24]. For example, if the signal-to-noise ratio was less than 0.5, it was excluded from the analysis. If the signal-to-noise ratio was greater than 0.5 but less than 2, it was categorized as “weak” and down-weighted. Also, when the peak values of a species were not reproduced by the model or the coefficient of determination (r^2^) was less than 0.7, that species was down-weighted to the “weak” category. For the outdoor PM_2.5_ PMF analysis, the elements that were labeled as weak consisted of molybdenum, phosphorus, nickel, copper, and zinc. The elements that were labeled as bad consisted of strontium, silver, indium, tin, neodymium, samarium, sodium, magnesium, aluminum, titanium, chromium, and cobalt. For the indoor PM_2.5_ PMF analysis, the elements that were labeled as weak consisted of EC, molybdenum, lead, phosphorus, calcium, nickel, and zinc. The elements that were labeled as bad consisted of strontium, silver, indium, tin, antimony, neodymium, samarium, uranium, sodium, magnesium, aluminum, titanium, chromium, and cobalt. For the indoor and outdoor models, respectively, indoor and outdoor PM_2.5_ concentrations were down-weighted to weak and included as a total variable to determine the source contributions to the daily mass concentrations. The optimal solution was determined by multiple model runs to examine the effect of the number of source factors on the correlation coefficients between the measured and modeled species, Q values (the goodness-of-fit parameter), residuals, and results from bootstrap runs, which aim to minimize factor swapping and determine the rotational ambiguity in a PMF solution by assessing the largest range of source profile values without an appreciable increase in the Q-value. The stability of the solution was also evaluated using bootstrap (BS), displacement (DISP), and bootstrap-displacement (BS-DISP, hybrid). Per recommendations in the PMF user guide, 100 BS runs with a default minimum R-value of 0.6 were performed to ensure the robustness of the results. The results from the bootstrapping work are summarized in Supplement Table S1. For each of the models (indoor and outdoor), BS and the base model displacement Error (DISP) were determined. For the indoor model, the BS-DISP diagnostics showed that 96% of the cases were accepted, with the largest decrease in Q-value. For the outdoor model, the BS-DISP showed that 100% of the cases were accepted, with the largest decrease in Q-value.

In order to determine statistical distribution differences in outdoor/indoor PM_2.5_ concentrations, their compositions, and factor contributions among the three intervention scenarios, the Friedman repeated measures analysis of variance was used. If the repeated ANOVA test showed a statistically significant difference, then post hoc comparisons via the Tukey test were completed. SigmaPlot (version 11.0, Systat Software Inc., San Jose, CA, USA) was used to perform these analyses (*p* < 0.001 indicates statistical significance).

## 3. Results and Discussion

The mean outdoor PM_2.5_ concentration across all exposure periods was 9.3 ± 4.1 µg/m^3^. The mean indoor PM_2.5_ concentrations during the sham, LE, and HE scenarios were 17.5 ± 16.9 µg/m^3^, 8.4 ± 5.4 µg/m^3^, and 7.0 ± 4.5 µg/m^3^, respectively [20]. The mean OC, EC, and elemental concentrations from the outdoor and indoor PM_2.5_ samples based on sham, LE, and HE filtration scenarios are presented in Table 1. As we observed in the PM_2.5_ mass concentrations, other major constituents, such as OC and EC, showed similar trends. For example, the mean indoor OC concentrations were reduced from 7.8 ± 8.4 µg/m^3^ (sham) to 3.5 ± 3.2 µg/m^3^ (LE) and 3.1 ± 2.2 µg/m^3^ (HE), and the reductions of HE and LE were not significantly different. Table 1 also shows the percent reduction of each constituent of indoor PM_2.5_ for the LE and HE air filtration interventions relative to sham. As anticipated, HEPA-type and true HEPA PAFs provided similar mitigation effectiveness for each constituent of indoor PM_2.5_. The mean negative percent reductions for P and Al in the HE scenarios are likely due to sampling errors.

### 3.1. Major Sources of Outdoor PM_2.5_

**Figure 1:**
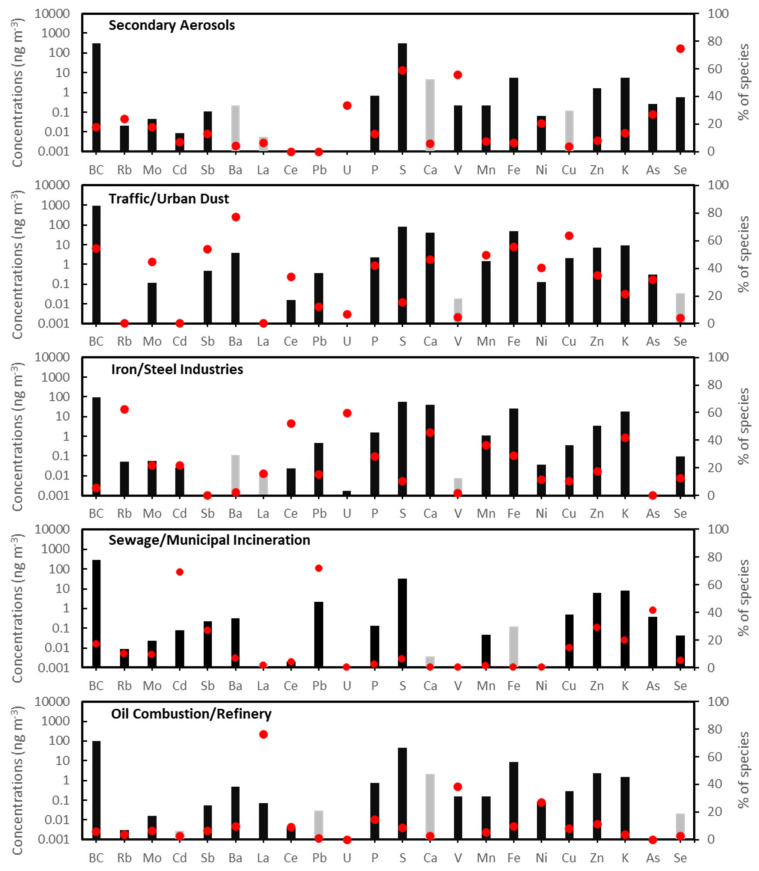
Factor profiles resolved for sources of outdoor PM_2.5_ based on PMF analysis of 257 outdoor filter samples collected from 2014 to 2016 (Black bars: significant based on the fifth percentile of the bootstrap uncertainty distribution analysis). (Gray bars: not significant/high uncertainty). Red points represent the percentage distribution of each species across the source factors.

**Figure 2:**
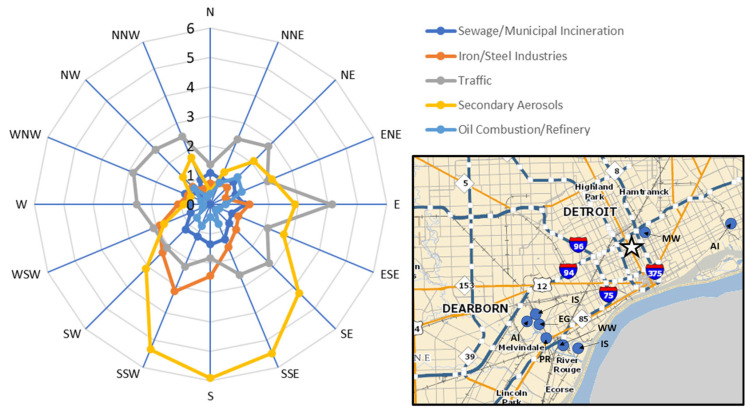
Average factor contributions (in µg/m^3^) to outdoor PM_2.5_ vs. wind direction. A Detroit area map showing the location of this study site (indicated by a star) and major industrial sources for PM_2.5_ [26,27] (AI: auto industries, PR: petroleum refineries, EG: energy generation, IS: iron/steel industries, MW: municipal waste incinerator, and WW: waste water treatment and sludge incinerator).

For outdoor PM_2.5_, PMF extracted five major source factors, including secondary aerosols, traffic/urban dust, iron/steel industries, sewage/municipal incineration, and oil combustion/refinery, and resolved factor profiles are shown in Figure 1. Figure 2 shows a map of the Detroit area, highlighting this study site and some of the major PM_2.5_ point and mobile sources in Wayne County [26,27]. It also shows wind rose plots of time-averaged PMF factor contributions as a function of wind direction for the outdoor PM_2.5_ data as observed at the community study site.

The first outdoor source contained the highest loadings of S, Se, and V. This factor, identified as secondary aerosols, is likely emissions from Midwest regional coal-fired utility boilers/power plants, as they have previously been associated with the highest contributions to sulfate due to gas-to-particle conversion of SO_2_ to sulfate via photochemical reactions [25,28]. This study site was impacted by multiple regional coal-fired power plants extending from the lower Great Lakes to the Ohio River Valley, and contributions of the secondary aerosol factor were elevated when the wind was from the south (Figure 2). The high loading of V suggests that this factor may also contain oil combustion and ship emissions since the Detroit River carries commercial shipping traffic. Previous studies have reported that elevated ambient V and Ni concentrations and V/Ni ratios between 2.5 and approximately 3.5 were linked to PM emitted by shipping [29,30]. However, for the present study, the average ratio of V/Ni in outdoor PM_2.5_ samples was only 1.7.

The second outdoor source was identified as traffic/urban dust. This factor had the highest loadings of BC as well as high loadings of Ba, Mn, Fe, Cu, Ca, Sb, and Zn. Previous studies have indicated that BC as well as these elements are tracers for road traffic emissions [31,32,33]. In addition, traffic sources commonly include a mixture of tailpipe emissions from gasoline/diesel engines, brake wear, and road dust [34,35]. The high loadings of Sb, Ba, and Cu may be vehicle-derived metals from brake/tire wear, and Zn may be from tire wear and lubricating oil [23,36,37,38]. High loadings of crustal elements, including Ca and Fe, suggest that this factor included suspended road/urban dust. The contribution from this factor varied relatively little with wind direction, which is consistent with this study community’s location in an area that is surrounded by major interstate highways (I-375, I-75, and I94) and state highways (M-1 and M-10) in Detroit.

The third source had high loadings of Rb, Ce, Mn, and Fe,, and was identified as iron/steel industries [39,40,41]. The wind rose plots of the factor contributions show that the highest iron/steel industry contribution was associated with south-southwesterly winds. The 2014 EPA National Emissions Inventory reported multiple large steel industries near this study site, including AK Steel-Dearborn Works (200 tons of PM_2.5_ emissions) and US Steel-Great Lakes Works (218 tons of PM_2.5_ emissions), and this wind plot further confirms probable locations of large iron/steel industries [26].

The fourth source was identified as sewage/municipal incineration based on high concentrations of Cd, Pb, and As. Pb emissions from waste incineration have been extensively studied [42,43,44], and high As levels can be related to anthropogenic waste and industrial activities such as sewage sludge incineration [45,46]. Prior studies have reported that refuse incineration is a major source of atmospheric Cd [47,48].

**Figure 3:**
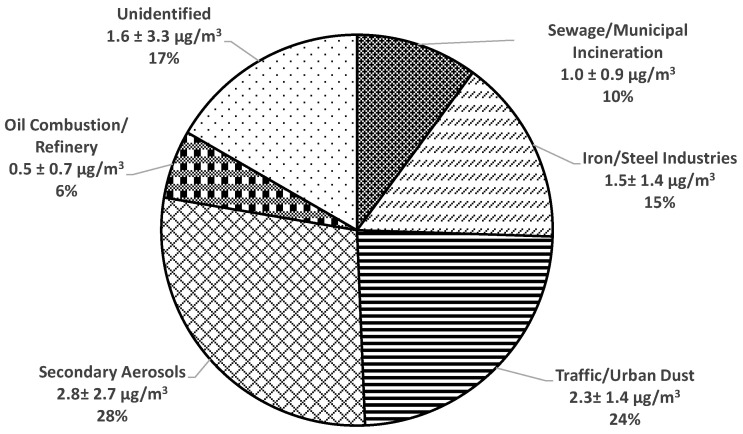
Average factor contributions to outdoor PM_2.5_ during the intervention periods from 2014 to 2016.

The fifth source had high loadings of La, V, and Ni. These elements are common tracers for oil combustion sources and refineries [49,50]. Figure 3 shows the reconstructed outdoor PM_2.5_ mass from all sources identified by PMF. The contribution from coal/secondary sulfate (28%) was the highest, followed by traffic/road dust sources (24%), iron/steel manufacturing (15%), sewage/municipal incineration (10%), and oil combustion/refinery (6%). Outdoor PM_2.5_ from unidentified sources was estimated to be 17% by calculating the difference between the reconstructed outdoor PM_2.5_ mass from all identified sources and the measured outdoor PM_2.5_. Previous studies have reported that unidentified PM sources may be partly due to PM measurements that include non-solid constituents, such as liquid water retained on soluble constituents during filter weighing and organic vapors adsorbed on quartz fiber filters [51].

### 3.2. Major Sources of Indoor PM_2.5_

**Figure 4:**
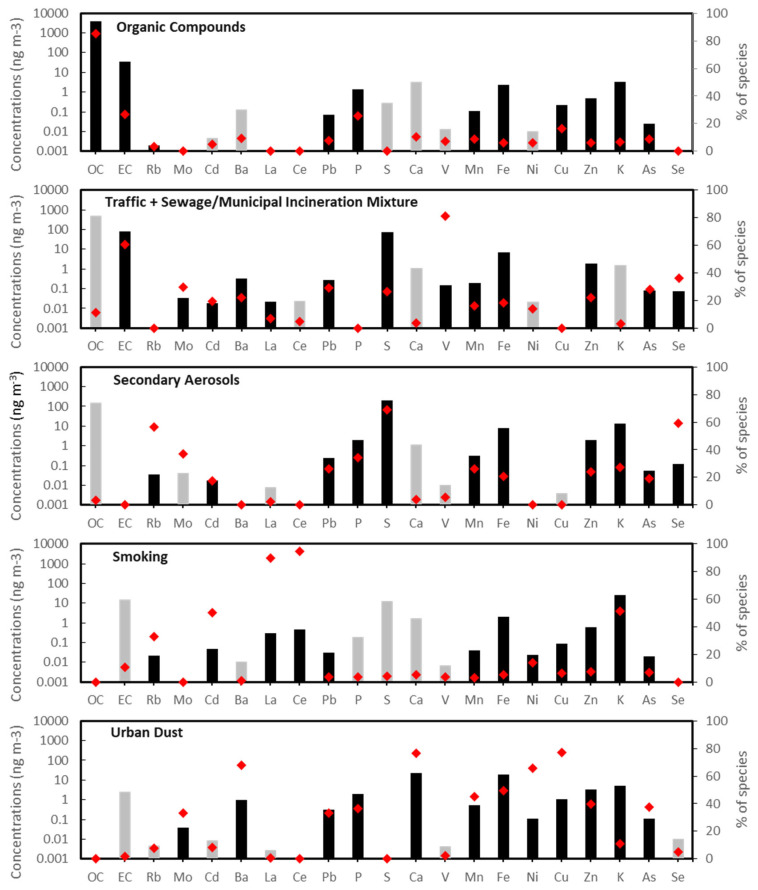
Factor profiles resolved for sources of indoor PM_2.5_ based on PMF analysis of 358 indoor filter samples collected from 2014 to 2016 (Black bars: significant based on the fifth percentile of the bootstrap uncertainty distribution analysis). (Gray bars: not significant/high uncertainty). Red points represent the percentage distribution of each species across the source factors.

**Figure 5:**
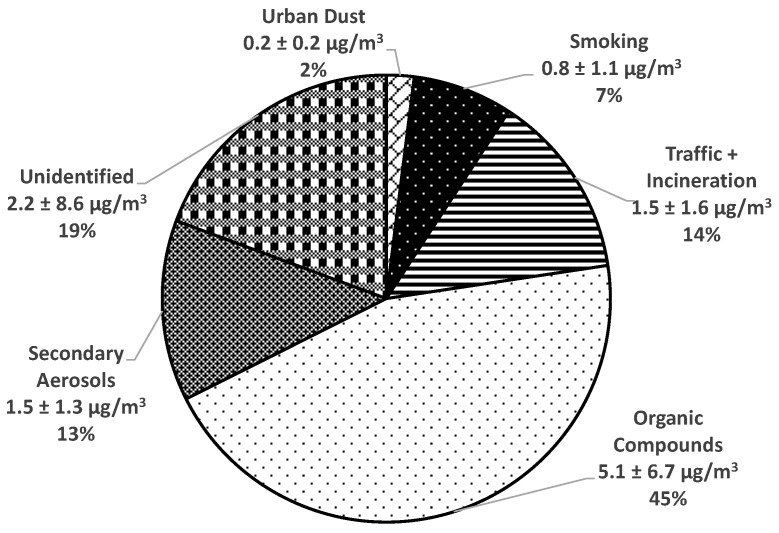
Average factor contributions to indoor PM_2.5_ during the intervention periods from 2014 to 2016.

Factor profiles resolved for five major sources of indoor PM_2.5_ based on PMF analysis of 358 indoor filter samples are shown in Figure 4. These sources included organic compounds (45%), traffic + sewage/municipal incineration (14%), secondary aerosols (13%), smoking (7%), and urban dust (2%) (Figure 5). Indoor PM_2.5_ from unidentified sources was estimated to be 19% by calculating the difference between the reconstructed indoor PM_2.5_ mass from all identified sources and the measured indoor PM_2.5_. The first source was identified as organic compounds based on the highest loadings of OC. As other studies have reported, the organic compound factor is attributable to routine human activities at home. First, it has been well documented that cooking activities have been linked to increased concentrations of organic compounds [52,53]. Second, although household cleaning and vacuuming activities are generally intended to remove dust and biological aerosols, they may result in the resuspension and redistribution of aerosols and dust. Other studies have reported that household products and building materials (e.g., cleaning products, carpets, cosmetics) were some of the major sources contributing to VOCs in indoor samples [54].

The second source had the highest concentrations of S (69%) and Se (59%). As described under “Major Sources of Outdoor PM_2.5_”, this source is commonly recognized as secondary aerosols. The third source had high loadings of EC, Ba, Mo, Pb, Fe, V, Zn, As, and Se and was identified as a mixture of traffic and incineration. The fourth source was identified as smoking based on the high loadings of Cd (50%), Ce (95%), La (90%), and K (52%). Previous studies have reported that high concentrations of Ce and La were found in indoor sites with tobacco smoking activity [55,56]. Cd, being present in tobacco in high concentrations, is a well-documented marker for smoking [55,57]. K is often associated with tobacco and wood combustion [33,58], but this study site did not have any fireplaces for wood combustion. The fifth source was identified as urban dust. This source primarily consisted of moderate loadings of many elements, including Ba (68%), Ca (76%), Mn (45%), Fe (49%), Ni (66%), Cu (77%), and Zn (40%). Previous studies have reported that urban dust is represented by high loadings of Fe, Mn, Cu, Zn, and Ca [35,59].

### 3.3. Effectiveness of PAFs against Outdoor PM_2.5_ Infiltration

Previous studies have reported that the use of sulfur or sulfate as an outdoor PM_2.5_ tracer is the most common method for estimating PM_2.5_ infiltration efficiency, which is the fraction of the outdoor concentration that penetrates indoors [60,61]. Sulfur is a useful tracer because it has few indoor sources, and thus the indoor/outdoor sulfur ratio provides a good estimate of infiltration efficiency for PM_2.5_. Based on this, the secondary aerosol factor that has the highest loading of sulfur was used to estimate PM_2.5_ infiltration efficiency for this study.

Table 2 shows, for each intervention scenario, the average concentrations of outdoor and indoor PM_2.5_ and outdoor and indoor secondary aerosols, according to the PMF analysis described above. There were no statistically significant differences in outdoor PM_2.5_ and outdoor secondary aerosols among the intervention scenarios. However, for both indoor PM_2.5_ and indoor secondary aerosols, comparison of the LE and HE concentrations against the no-filter (sham) concentration showed significant differences. The average infiltration rates based on secondary aerosol were 79 ± 24% for no-filter (sham), 61 ± 32% for LE filters, and 51 ± 34% for HE filters, respectively, and the PAFs were able to significantly reduce infiltration of outdoor PM_2.5_.

### 3.4. Limitations of This Study

While we were able to collect indoor PM_2.5_ samples on both quartz and Teflon filters, we were able to collect only one Teflon filter sample each day for outdoor PM_2.5_ during this study. The lack of quartz-filtered outdoor samples precluded OC analysis and likely increased uncertainty for factor profiles and contributions.

Because the subjects in this study all lived in the same residential facility, many variables were controlled for, such as apartment floorplan and size, stove type (electric), and proximity to streets and industrial sources of PM_2.5_. However, since this study did not restrict activities, each subject engaged in activities such as leaving windows open, cooking meals, and using cleaning supplies, all of which resulted in microenvironments with diverse characteristics and are likely responsible for the “unidentified” source shown in Figure 5.

Another limitation of this study was that it did not quantify potential time-varying factors such as gaseous pollutants (e.g., ozone and nitrogen oxides). While the concentrations of gaseous pollutants are not affected by PAFs, examining these potential confounding factors may be important for future studies. In addition, a PM parameter such as ultrafine fraction (i.e., ultrafine PM, particles smaller than 100 nm) was not measured in this study. As some studies report that long-term exposure to ultrafine particles is associated with an increased risk of CV morbidity and mortality [62,63], the evaluation of such parameters may prove useful.

Finally, although this air quality intervention study has shown that, compared to sham, air filtration for 3 days using PAFs decreased 3-day average brachial systolic blood pressure by 3.2 mmHg [19], we were not able to determine which reductions in major PM_2.5_ sources (if any) were associated with the reductions in systolic blood pressure since this study was not powered for that secondary analysis. Further study is needed to investigate whether reductions of any specific indoor or outdoor PM_2.5_ sources via this intervention are especially closely related to blood pressure reductions.

## 4. Conclusions

The results from this study show that intervention with PAFs was able to significantly decrease indoor PM_2.5_ concentrations from both outdoor and indoor major sources in Detroit, Michigan. As we previously reported, this air quality intervention study has also shown that the use of PAFs decreased 3-day average brachial systolic blood pressure by 3.2 mmHg. To our knowledge, this is the first study that evaluated what types of outdoor and indoor PM_2.5_ sources the subjects were exposed to in an intervention study in which a significant positive health effect was observed. Our detailed chemical characterization and source apportionment revealed that the infiltration of outdoor PM_2.5_ without any PAFs was about 79%. The use of HEPA-type and true HEPA air filtration has been shown to reduce outdoor PM_2.5_ infiltration rates from 79% to 61% and 51%, respectively. Such reductions may be useful for protecting susceptible populations, including older adults and people with pre-existing diseases. Furthermore, when extreme air pollution events such as wildfires occur, PAFs may provide useful protection for larger portions of the population in affected areas. The results of this study provide insights into what degree of air quality improvement is possible through the use of commercially available PAFs in one of the most vulnerable communities. Because this study was limited to one residential facility, future studies are warranted to improve our understanding of how to optimally implement this personal-level intervention in various other settings, including single-family homes and locations with different demographics and outdoor sources.

## Figures and Tables

**Table 1:** Average mass concentrations and chemical composition of PM_2.5_ and percent reduction of each constituent (relative to indoor sham) during the three different intervention scenarios.

	Outdoor	Indoor—Sham	Indoor—LE	Indoor—HE
PM_2.5_ (µg/m^3^)	9.3 ± 4.1	17.5 ± 16.9	8.4 ± 5.4 (52%)	7.0 ± 4.5 (60%)
OC (µg/m^3^)	-	7.8 ± 8.4	3.5 ± 3.2 (55%)	3.1 ± 2.2 (61%)
EC (µg/m^3^)	1.9 ± 0.9	0.3 ± 0.1	0.1 ± 0.0 (43%)	0.1 ± 0.2 (44%)
Element (ng/m^3^)				
Rb	0.10 ± 0.09	0.11 ± 0.09	0.06 ± 0.04 (39%)	0.07 ± 0.15 (32%)
Mo	0.40 ± 0.44	0.31 ± 0.37	0.18 ± 0.23 (40%)	0.14 ± 0.17 (54%)
Cd	0.15 ± 0.28	0.18 ± 0.14	0.10 ± 0.08 (41%)	0.09 ± 0.07 (46%)
Sb	0.97 ± 0.64	0.46 ± 0.30	0.30 ± 0.26 (35%)	0.24 ± 0.20 (48%)
Ba	5.89 ± 7.06	2.49 ± 2.59	1.55 ± 2.62 (38%)	1.34 ± 1.14 (46%)
La	0.10 ± 0.13	0.65 ± 1.18	0.34 ± 0.50 (48%)	0.35 ± 0.68 (46%)
Ce	0.06 ± 0.07	0.93 ± 1.76	0.46 ± 0.77 (51%)	0.47 ± 0.91 (50%)
Pb	4.09 ± 5.31	2.01 ± 1.65	1.41 ± 1.69 (30%)	0.94 ± 0.85 (53%)
U	3.21 × 10^−3^ ± 2.76 × 10^−3^	1.43 × 10^−3^ ± 1.98 × 10^−3^	1.30 × 10^−3^ ± 2.72 × 10^−3^ (9%)	9.58 × 10^−4^ ± 1.12 × 10^−3^ (33%)
Na	27.5 ± 31.26	66.34 ± 157.42	39.33 ± 49.59 (41%)	87.29 ± 273.68 (−32%)
Mg	27.06 ± 20.40	34.97 ± 215.13	18.24 ± 51.60 (48%)	9.10 ± 6.57 (74%)
Al	10.04 ± 28.67	17.78 ± 16.92	16.62 ± 26.89 (7%)	18.27 ± 37.28 (−3%)
P	7.05 ± 6.13	22.26 ± 88.66	12.33 ± 17.63 (45%)	12.23 ± 21.00 (45%)
S	633.70 ± 421.50	487.27 ± 421.37	276.72 ± 216.77 (43%)	215.33 ± 159.88 (56%)
Ca	99.20 ± 68.63	149.68 ± 874.56	77.34 ± 197.94 (48%)	46.53 ± 54.40 (69%)
V	0.52 ± 0.47	0.30 ± 0.24	0.18 ± 0.18 (42%)	0.14 ± 0.14 (53%)
Mn	3.34 ± 2.01	1.83 ± 0.95	1.18 ± 0.91 (36%)	1.03 ± 0.77 (44%)
Fe	96.03 ± 67.43	58.01 ± 45.91	43.82 ± 53.37 (24%)	34.32 ± 26.49 (41%)
Co	0.02 ± 0.02	0.02 ± 0.03	0.01 ± 0.01 (38%)	0.01 ± 0.01 (43%)
Ni	0.53 ± 0.80	0.35 ± 0.46	0.19 ± 0.21 (46%)	0.21 ± 0.22 (40%)
Cu	3.93 ± 4.27	2.62 ± 2.65	1.68 ± 2.06 (36%)	1.62 ± 2.96 (38%)
Zn	34.95 ± 49.28	32.11 ± 48.10	11.84 ± 15.79 (63%)	10.81 ± 15.75 (66%)
K	63.73 ± 155.72	82.82 ± 77.57	51.22 ± 61.82 (38%)	47.45 ± 61.42 (43%)
As	1.15 ± 0.96	0.55 ± 0.39	0.33 ± 0.25 (39%)	0.24 ± 0.21 (56%)
Se	1.01 ± 1.15	0.44 ± 1.48	0.24 ± 0.37 (46%)	0.18 ± 0.29 (58%)

Concentrations are in mean ± standard deviation. The percent reduction in the concentration of each constituent (relative to indoor sham) for each of the air filtration interventions is listed in parentheses.

**Table 2:** Comparison of average concentrations of outdoor and indoor PM_2.5_ and outdoor and indoor secondary aerosols based on PMF analysis.

Filtration Scenarios	Outdoor PM_2.5_ (µg/m^3^)	Outdoor Secondary Aerosols (µg/m^3^)	Indoor PM_2.5_ (µg/m^3^)	Indoor Secondary Aerosols (µg/m^3^)
Sham (no filter)	9.0 ± 3.7	2.3 ± 1.9	15.8 ± 12.2	2.0 ± 1.5
LE	9.1 ± 4.0	2.5 ± 2.3	8.4 ± 5.5	1.4 ± 1.3
HE	9.7 ± 4.5	2.6 ± 2.3	7.0 ± 4.5	1.1 ± 1.1

Bolding indicates a statistically significant difference (*p* < 0.001) based on comparison to the sham scenario.

## Data Availability

Data sharing is possible upon request.

## Supplementary Materials

The following supporting information can be downloaded at: https://www.mdpi.com/article/10.3390/toxics11121019/s1, Table S1: Outdoor and indoor base error estimations from creating factor profiles resolved from the positive matrix factorization analysis.
