# Supplementary material for: Reduction of Outdoor and Indoor PM2.5 Source Contributions via Portable Air Filtration Systems in a Senior Residential Facility in Detroit, Michigan
Source: Toxics. 2023 Dec 14;11(12):1019. doi: 10.3390/toxics11121019 (PMC10748160; doi:10.3390/toxics11121019)
Supplement: Supplementary file 1 [file toxics-11-01019-s001.zip › toxics-2741464-supplementary.pdf]

Supplemental Materials:

Table S1. Outdoor and indoor base error estimations from creating factor profiles resolved from the positive matrix factorization analysis.

Outdoor BS Mapping:						
	Sewage/Municipal Incineration	Iron/Steel Industries	Traffic	Secondary Aerosols	Oil Combustion Refinery	Unmapped
Boot Factor 1	96	2	1	1	0	0
Boot Factor 2	0	100	0	0	0	0
Boot Factor 3	0	0	100	0	0	0
Boot Factor 4	0	0	0	100	0	0
Boot Factor 5	0	0	0	0	100	0

Indoor BS Mapping:						
	Iron/Steel Industries/Urban Dust	Smoking	Traffic	Organic Compounds	Secondary Aerosols	Unmapped
Boot Factor 1	98	0	0	0	0	2
Boot Factor 2	0	98	0	0	0	2
Boot Factor 3	0	0	95	0	3	2
Boot Factor 4	1	3	0	93	1	2
Boot Factor 5	0	5	0	0	93	2
